# Olfactory dysfunction as a post-infectious symptom of SARS-CoV-2 infection

**DOI:** 10.1016/j.amsu.2022.103352

**Published:** 2022-02-11

**Authors:** Banw Anwar Othman, Sazan Qadir Maulud, Paywast Jamal Jalal, Saman Muhsin Abdulkareem, Jivan Qasim Ahmed, Manish Dhawan, Priyanka, Om Prakash Choudhary

**Affiliations:** a Department of Pharmacognosy, College of Pharmacy, University of Hawler Medicine, Iraq; b Department of Biology, College of Education, Salahaddin University-Erbil, Iraq; c Department of Biology, College of Science, University of Sulaimani, Iraq; d Department of Pathology and Microbiology, University of Duhok, Iraq; e Department of Microbiology, Punjab Agricultural University, Ludhiana, 141004, Punjab, India; f The Trafford Group of Colleges, Manchester, WA14 5PQ, UK; g Independent Researcher, 07, Type IV Quarter, College of Veterinary Sciences and Animal Husbandry, Central Agricultural University (I), Selesih, Aizawl, 796015, Mizoram, India; h Department of Veterinary Anatomy and Histology, College of Veterinary Sciences and Animal Husbandry, Central Agricultural University (I), Selesih, Aizawl, 796015, Mizoram, India

**Keywords:** SARS-CoV-2, COVID-19, Olfactory dysfunction, Smell loss, Taste loss

## Abstract

The unexpected onset smell and taste disability was being recognized as a COVID-19 related symptom. Loss of smell might occur alone or be followed by other COVID-19 symptoms, such as a dry cough, fever, headache, and shortness of breath. Other virus infections have been linked to anosmia (parainfluenza, rhinovirus, SARS, and others), affecting up to 20% of the adult population, which is much less common than SARS-CoV-2 infection. A hypothesis about the pathophysiology of post-infectious olfactory loss is that viruses could make an inflammatory response of the nasal mucosa or directly damage the olfactory neuroepithelium. However, in patients with COVID-19, loss of smell may occur without other rhino logic symptoms or suggestive nasal inflammation. According to evidence, anosmia-related SARS-CoV-2 could be a new viral syndrome unique to COVID-19. Furthermore, through experimental intranasal inoculation in mice, SARS-CoV-2 can be inoculated into the olfactory neural circuitry. This disease has not had the required focus, most likely because it is not life-threatening in and of itself. Though patients' quality of living is significantly reduced as their olfactory ability is lost, resulting in lowering and inadequate appetite, excessive or unbalanced food consumption, as well as an overall sense of insecurity. This review aims to give a quick overview of the latest epidemiological research, pathological mechanisms for the dysfunction of smell, and taste in patients infected with SARS-CoV-2. In addition, the initial diagnosis and treatment options for dysfunction are also discussed.

## Highlights

• The pathological mechanism of SARS-CoV-2 infection in olfactory dysfunction.
• Pathognomonic symptoms of sinonasal impairment in COVID-19 patients.
• Treatment of olfactory dysfunction after SARS-CoV-2 infection.

## 1 Introduction

On December 31, 2019, a novel coronavirus (COVID-19) was discovered in Wuhan, Hubei Province, China, as a severe respiratory disease of humans [1,2]. SARS-CoV-2, like SARS-CoV and MERS-CoV, is a member of the *Coronaviridae* family. That has a single-stranded RNA genome (a positive-sense single-stranded RNA, enveloped virus). The genome size is the largest among RNA viruses [3]. The invasion of coronavirus to the target cells is intermediated by a spike glycoproteins' transmembrane (S), which is made up of two subunits: S1, which has responsibility for attaching to hosts' cell receptors, and S2, which is responsible for fusion with the hosts' cell membrane. Since cleavage by a particular protease, these subunits will stay in a prefusion conformation. The receptor-binding (RBD) domains are found in the distal S1 subunit, mainly active in recognition of angiotensin-converting enzyme 2 (ACE2) [4,5]. The host protease will further cleave S protein and activate membrane fusion. Because of the peripheral site of S proteins, they are a primary focus for neutralizing antibodies and emerging new treatments [6,7]. ACE2 receptor is a functional SARS-CoV-2 receptor with a broad range in the human body; the higher expression is in the nasal mucosa, lung parenchyma of the lung, and gastrointestinal tract. Also, ACE2 receptors have been found on glial cells and neurons [8]. A mouse model showed that SARS-CoV-2 enters via the olfactory bulb [9] (see Table 1).

**Table 1:** Medicines that may cause smell disorders.

Group	Examples of substances
Antibiotics	Streptomycin
Antirheumatic drugs	Gold
Antihypertonic drugs	Diltiazem, nifedipine
Antidepressants	Amitriptyline
Chemotherapeutic drugs	Methotrexate
Local anesthetics	Cocaine
Opioids	Remifentanil, morphin
Psychopharmaceuticals	Amphetamines, alcohols
Sympathomimetics	Chronic use of local vasoconstrictive substances
Others	Sildenafil

Moreover, besides being classified as respiratory viruses, coronaviruses are considered to be neurotropic and neuro-invasive in addition to being epitheliotropic [4,10]. As this pandemic in post-viral olfactory dysfunction (PVOD) emerges, it necessitates renewed interest and evidence-based treatments. It is impossible to predict PVOD's natural history [11]. Although other coronaviruses are shown to cause neurological invasiveness, the concern is whether SARS-CoV-2 enters the brain via the neuroepithelium [12]. In COVID-19, the nasal cavity plays a significant part. The nasal cavity is most likely the site of viral entry and a hotbed of viral replication [13]. For a long time, olfactory conditions have been linked to viral upper respiratory tract infections such as common flu, including influenza and parainfluenza viruses, rhinoviruses, and other coronaviruses found in endemic areas [14,15]. Also, taste disturbances have been reported throughout and after a respiratory viral infection [16]. Thus, SARS-CoV-2 causes anosmia and ageusia, but the precise pathogenesis is unknown. However, central nervous system (CNS) involvement and nasal epithelium damage caused by microorganisms might be plausible causes of anosmia [17,18]. There are studies presented that coronavirus through olfactory nerves or epithelium of olfactory system by neuro invasive mechanism might infect the human CNS, COVID-19 may induce dysosmia and dysgeusia by damaging the trigeminal and olfactory nerves, SARS-CoV-2 RNA was recently discovered in the cerebral fluid, indicating that the virus is neuro invasive [19]. An additional effect of COVID-19 infection might be a reduction in the sensitivity of sensory neurons [3]. Sinonasal pathophysiology has been uniquely brought to the forefront with important roles in infection, transmission, and pathognomonic symptomatology that may identify infected individuals [20]. During the emergence of COVID -19, authors have reported a sudden increase in anosmia in patients infected by this virus. Mao et al. originally recorded this finding in 2020.

Meanwhile, in several subjective studies, new-onset olfactory or gustatory dysfunction has been identified in combination with other well-known symptoms of COVID-19 infection [13]. Moein et al. used the University of Pennsylvania Smell Identification Test Moein et al.; Hyposmia or anosmia affected 98% of the COVID-19 cohort. Kurdistan region of Iraq was not out of this viral pandemic; in March 2020, the first case was recorded according to an epidemiological study that also indicated infected patients' symptoms [21]. The most common symptoms documented in almost 79% of the cases were patients suffering from a dry cough, fever, fatigue, and breathing difficulty were also recorded frequently [21]. The Centers for Disease Control and Prevention (CDC) also added a new disability of taste or smell to the list of signs that can occur 2–14 days after COVID-19 exposure [1].

One of the five primary humans' senses is olfaction, which performs a multitude of vital health-related functions such as the detection of health risks such as fire or poisonous gases and the capacity to enjoy food does have psychosocial implications [11], which might have a detrimental impact on one's quality of life [22]. It has been reported that olfactory disorder is one of the most popular issues post COVID-19 infection [23]. Numerous pharmacological therapies for olfactory dysfunction of various etiologies have been tried, with mixed results, for example, zinc gluconate, intranasal insulin, minocycline, vitamin A, vitamin B, Ginkgo biloba, caroverine and corticosteroids. Olfactory training (OT) is an effective therapy for OD caused by various factors and is currently considered the most effective treatment for OD [22]. The capacity of the olfactory neuron system to restore damaged olfactory neurons in the olfactory epithelium is well known, and it has offered that olfactory training might enhance this regeneration [11,22,24]. The present study aims to illustrate the relationship between OD and COVID-19. Nevertheless, it enhances our understanding of olfactory circuits' dysfunction physiology and offers additional information on the causes of odour issues.

### 1.1 Olfactory physiology

**Fig. 1:**
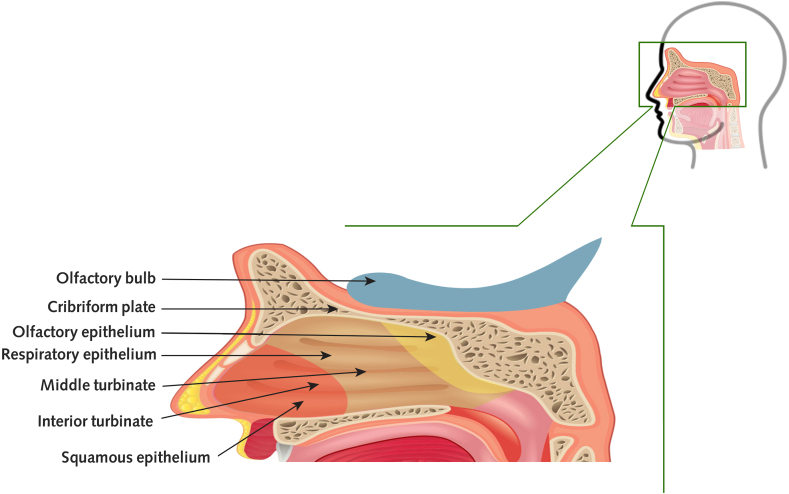
A sagittal view of the respiratory tract beside olfactory, respiratory, and squamous mucosa is shown in this diagram.

On the rooftop of the nasal cavity, the olfactory epithelium (OE) (Fig. 1) is situated. As a result, it is not in the primary airflow stream of breathing [16]. The olfactory cleft is a short passageway that allows for both ortho-and retronasal airflow; scents can reach the olfactory cleft by sniffing via the nose, although it occurs when eating or drinking, via the nasopharynx by going retro nasally into the nose [25]. Because of the olfactory neuroepithelium's unique position, the respiratory control the effects of the local smell concentration in specific ways [16,26], and gives essential environmental information, which it's the reason behind the substantial neural circuitry has been dedicated to processing olfaction and multisensory integration [25].

The epithelial layer contains at least five types of cells: olfactory sensory neurons (OSNs), sustentacular cells, microvillar cells, duct cells of the olfactory Bowman's glands, and basal cells [27]. Odorant receptors on the cilia of the olfactory sensory neurons sense the odorants. The odorant receptor is composed of coupled G-protein receptors that will activate Golf. By Golf activation, adenylyl cyclase will stimulate after it comes to the formation of cyclic adenosine monophosphate. Adenosine monophosphate results in action potential via the opening of chloride channels and then an efflux of chloride ions [28]. OSNs of the olfactory bulb are bipolar neurons with axons that form synapses. Also, they had many projections called dendrites that protrude out from the nasal cavity and are enclosed by sustentacular cells. Each dendritic knob has 10–30 cilia that emerge into the mucus layer [29]. Every OSN represents a distinct OR type and all OR-specific OSNs send their axons to the glomeruli, then in the olfactory bulb, they synapse with mitral and tufted cells. Second-order olfactory neurons (mitral and tufted cells) transmit their axons to a variety of olfactory locations in the central nervous system (CNS), which includes the anterior olfactory nucleus (AON), the olfactory tubercle, the piriform cortex (area 51), the amygdala, and the entorhinal cortex [28]. During each person's life, OSNs in the OE is constantly changing. Globose and horizontal basal cells are polymorphic, meaning they can produce all sorts of OE cells. A study in murine models with injured OE by intranasal lipopolysaccharide showed that steroids would inhibit regeneration of OSN [30]. Neuroblasts from the sub-granular zone of a dentate gyrus are used to regenerate neurons in the olfactory bulb [25,31].

### 1.2 Aetiology of the loss of smell

There are two types of odour loss quantitative; when a person's capacity to detect variations in the intensity of an odour is harmed, or qualitative, when the capacity to distinguish distinctions in odour quality is harmed. The word normosmia refers to the normal function of the olfactory; a decrease in smell called hyposmia as a quantitative disorder, and a total loss of odour is named anosmia [32]. The way we smell changes physiologically as we get older; although the human capacity to identify odours improves throughout life, it maximizes in the fourth decade; after the sixth decade, the ability to recognize and identify odours decreases dramatically [33]. OD is linked to various diseases, counting congenital causes, post-infectious disorders, sinonasal diseases, traumatic brain injuries, and neurodegenerative disorders. Few concrete observations explain the aetiology of smell loss; (olfactory bulb hypoplasia or aplasia) as in congenital causes (hypogonadotropic hypogonadism) in Kallmann syndrome and (absence of all or part of one X chromosome) as in Turner syndrome, the upper respiratory post-viral infection might be caused by a conductive and sensorineural/inflammatory disease in combination [34]. Like allergic "rhinitis and rhinosinusitis", sinonasal diseases might result in conductive and inflammatory conditions, which serve as anatomical obstacles to odorants entering the olfactory epithelium and receptors [35]. Hurtful head damages are sensorineural disorders due to the focus on initial stabilization and therapy. Patients and their carers frequently ignore sensorineural problems [36]. There are several more sources of odour problems such as exposure to toxic substances, psychiatric illnesses such as schizophrenia and depression, epilepsy or due to systemic diseases, for example, sarcoidosis, lupus erythematosus, as well as endocrine disorders like hypothyroidism, diabetes or isolated organ deficiencies as in renal failure and liver failure or cancerous growths such as esthesioneuroblastoma and other carcinomas of the intranasal cavity and brain tumours benign or malignant. Neurosurgical operations have been mentioned as untreatable causes of smell problems, radiation, medication intake shown in table [1] and, on rare occasions, ENT operations. Usually, the exact source of an odour problem cannot be determined [37].

### 1.3 Pathological mechanisms of smell dysfunction in SARS-CoV-2 infection

By using spike glycoprotein (S protein), which is the initial target for antibody neutralization, coronaviruses connect to the hosts' receptor and promote entry of the virus by membrane fusion. The strong virulence of SARS-CoV-2 S protein, which has significantly greater affinities than SARS-CoV and a lower thermostability factor than SARS-CoV, is indeed a vital aspect in the fast-rising spread of COVID-19 [38,39]. SARS-CoV-2 utilizes the human angiotensin-converting enzyme 2 (hACE2) as the receptor for host cell entrance; according to research, the transmembrane Serine Protease 2 (TMPRSS2) is used for S protein priming and activation, primarily by endocytosis. TMPRSS2 has a significant role in propagating various viruses, including influenza A and coronaviruses [40].

Due to various inflammatory reactions, many viruses produce transitory alterations in smell sense. The impairment of smell has been linked to previous coronavirus infections. However, that was a very unusual occurrence. Hwang reported a case of permanent anosmia in a female patient in 2006. People suffering from COVID-19 have reported in a recent study that has significantly recurrent olfactory loss in comparison with other influenza patients [41]. Loss of smell was observed in COVID-19 individuals who did not have additional coryza symptoms or substantial nasal irritation. This discovery is most likely the source of the problem. The possible mechanisms are: first, the straight damage of the virus on the neurons receptors in the olfactory epithelium will be damaged by the virus [42], and secondly, immune cells and cytokines are produced in excess or uncontrollably "cytokine storm" that has been begun in some patients that disturb the nervous system, including sensory organs of smell [43,44]. However, a different immune response in the nasal cavity is the plausible hypothesis: A more robust immune response is predicted to impair smell, but it may also avoid transmitting pathogens to deeper respiratory organs like the lungs. On the other hand, a weaker and more limited immune response may allow virus proliferation and migration to the lower respiratory system, with its recognized life-threatening implications [45]. Third, the presence of both protein receptors ACE2 and TMPRSS2 are essential for effective SARS-CoV-2 infection in humans in non-neural cells in the olfactory epithelium [39].

**Fig. 2:**
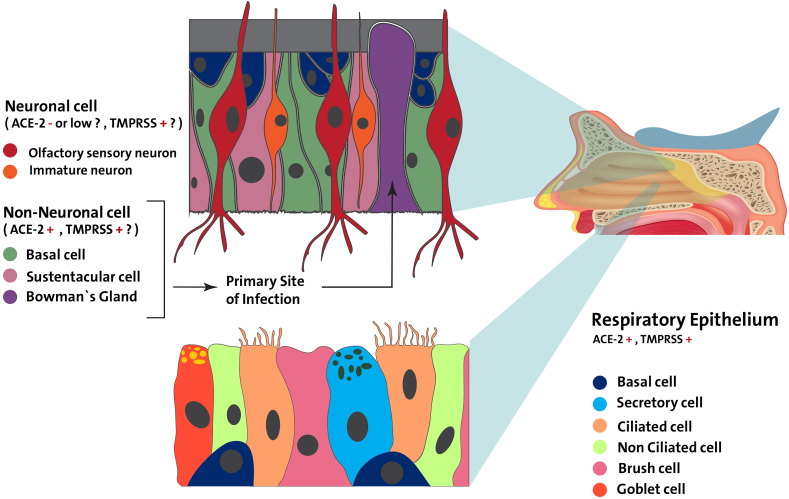
The expression of ACE-2 and TMPRSS is shown in a schematic showing the essential structure of the respiratory and olfactory epithelium.

Respiratory epithelium and sensory olfactory epithelium are two types of nasal epithelium, a multilayer structure that continually regenerates and contains neuronal and non-neuronal cells. In Butowt and Bilinska study, ACE2 expression in the non-neuronal olfactory epithelium has been strongly suggested. However, there is no clear evidence that could demonstrate the expression of this receptor in the neuronal olfactory epithelium [39]. TMPRS2 appears to have a greater expression level than ACE2 in both neuronal and non-neuronal cells [46]. A study reported that ACE2 and TMPRSS2 are two essential receptors involved in SARS-CoV-2 entrance that is expressed across the olfactory mucosa in both mouse and human species [42]. But these genes are not found in olfactory sensory neurons or olfactory bulb neurons, although they are found in supporting cells, stem cells, and perivascular cells (Fig. 2). Brann et al., 2020 [42] proposed that viruses may not directly invade olfactory sensory neurons, but they may infect supportively and stem cells in the olfactory epithelium. They suggested that anosmia in COVID-19 patients may be caused by initial infection of non-neutral cell types through; strong immune reaction result from the site of infection of supporting cells and vascular pericytes would results in alteration olfactory function, also supporting cells damage might have an indirect impact on olfactory sensory neuron transmission to the brain. In animal models, injury to sustentacular cells and Bowman's gland cells causes widespread structural injury to the whole olfactory epithelium, disrupting smell function [46].

**Fig. 3:**
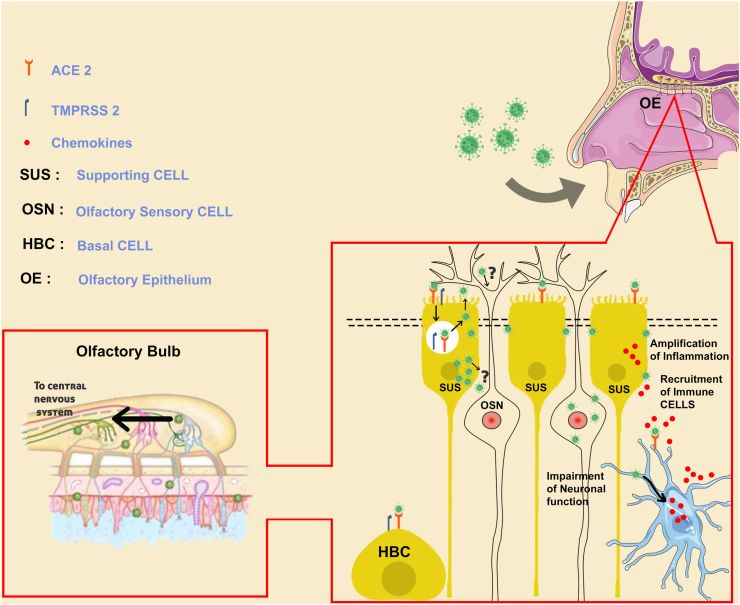
The olfactory epithelium serves as a neural path for SARS-CoV-2 infection.

Coronaviruses viruses can pass into the brain directly by arriving at the terminal of the peripheral nerves and then utilizing an active transport mechanism inside those cells to move toward and pass synapses reaching the CNS [47,48]. Alternatively, it will arrive directly into the cerebrospinal fluid through the nose as the nose is in connection with the CSF [49]. At the meantime, there are no evidence established whether SARS-CoV-2 will reach to the central nervous system or not. Alternatively, other coronaviruses, including SARS-CoV-1, have been found in human CSF and the brain [50]. SARS-CoV-2 and MERS-CoV were shown to enter the brain through olfactory neurons, the thalamus, and the brain stem in transnasally infected mice [51]. Coronaviruses might exploit retrograde neuronal transport from the lungs into the CNS via the vagal nerve afferents or enter the CNS through the gastrointestinal system as part of the brain-gut axis, including the vagus nerve [52]. One more possibility is that human coronavirus can enter the CNS via passing through the epithelium and entering the blood circulation or lymphatic system. Viruses can infect various myeloid cells to alter innate immunity and spread to further organs, including the CNS [53,54]. These concerns of entrance and penetration pathways would need to be addressed in the future for SARS-CoV-2. The majority of COVID-19 patients do not even have a nasal blockage or decreased airflow that results from mucosal swelling. According to one study, only 4% of individuals with documented olfactory dysfunction (OD) have nasal blockage [41]. This suggests that smell loss is caused by something other than rhinitis (mucosal membrane inflammation or irritation, nasal obstacle and discharge), for instance, injury to the olfactory system's peripheral and/or central components, as shown in (Fig. 3).

### 1.4 The method used to treat the loss of the sense of smell

#### 1.4.1 Drugs

According to a French Society of Otolaryngology paper, patients should avoid corticosteroids to treat SARS-CoV-2 infection [55]. On the other side, physicians commonly employ empirical oral steroids to manage anosmia to reduce swelling and irritation. Given the elevated risks of immunosuppression associated with these medicines, they believe tailored case management and therapy should be used. When intranasal steroids are used, the overall risk appears to be lowered. The severity of an upper respiratory tract viral infection is the critical issue in this case [56]. Nasal saline irrigations, followed by nasal and oral corticosteroids, have been the most often utilized therapy for olfactory impairment in SARS-CoV-2 affected individuals. According to Lechien et al., Doctors employed l-carnitine or essential vitamins and minerals to treat taste dysfunction [57].

#### 1.4.2 Olfactory training (OT)

Olfactory training is a potential therapy option for OD caused by various factors; OT is now the OD treatment with the most proof of effectiveness [58]. The ability of the nasal neuron system to repair injured olfactory neurons in the olfactory epithelium has been well established through It is thought that OT might help with regeneration. It might also have a role in influencing the central structure of the olfactory in the olfactory bulb and could make a change in connection with the brain [59]. Sniffing four distinct odours two times, a day for a suggested length of three months or longer is part of proven OT [22]. Throughout the context of smelling recovery in various kinds of smell loss, odour training is a straightforward, secure, and widely obtainable therapy [6,51,60]. In ten investigations, OT was found to enhance olfactory function. Greater odour concentrations & molecular weights, longer OT sessions, and a range of scents utilized for OT were shown to be the most effective in enhancing olfactory function [11]. Study results in the Medical University of Vienna showed that training enhances modifications in functional connectivity of the main olfactory areas [24].

#### 1.4.3 Zinc

Zinc (Zn) is a necessary micronutrient and the human body's second most abundant metal. Zn is essential for cell proliferation, differentiation, and survivability. About 10% of the human genome's Zn-finger motifs can bind Zn [61]. Zinc affects the function and structure of enzyme and transcriptional factors as it's a protein cofactor and acts as a second messenger [8]. Intracellular Zn has been demonstrated to suppress SARS-CoV RNA-dependent RNA polymerase elongation in infected cells in previous investigations with SARS-CoV [62]. In coronavirus-infected cells, ZN ions were also demonstrated to impede the proteolytic processing of replicase polyproteins [8].

## 2 Conclusion

Olfactory dysfunction could be induced by various factors and can occur due to a variety of diseases. In particular, lack of smell and weakening of smell are dependable primary signs of various clinical conditions, reaching from virus infections involving SARS-CoV-2, to neurodegenerative conditions, with Alzheimer's and Parkinson's diseases. Even though COVID-19 is essentially a pulmonary illness, many other organs in the body may be affected by COVID-19, involving CNS. The disease results in a multitude of symptoms such as headaches and a lack of taste and smell. Viruses and poisons might also enter the human brain throughout the olfactory structure, resulting in infections and severe neuroinflammatory responses in the CNS. Viruses were proven to be carried into the CNS via synaptic connections from the peripheral olfactory epithelium. They begin by concentrating on olfactory system parts, including the olfactory bulb, amygdala and others. It eventually reaches other structures that would cause disease-specific symptoms to appear. Research with long-term follow-up on individuals with unexpected anosmia is critical since this symptom might signal the beginnings of neuroinvasion, leading to persistent neurodegenerative disease. The pathogenic mechanisms are unknown; however, most probably due to the primary infectivity of non-neuronal epithelial cell types of olfactory will lead to damage to olfactory neurons. Because olfactory impairment can negatively affect a patient's quality of life, more clinical research is needed to determine the pathophysiology, outcome, and link between illness severity and olfactory impairment on a global scale. As a therapeutic, olfactory training may benefit some persons suffering from permanent smell loss after COVID-19.

## Provenance and peer review

Not commissioned, externally peer reviewed.

## Ethical approval

Not applicable.

## Consent

Not applicable.

## Author contribution

**Banw Anwar Othman:** Conceptualization, Data Curation, Visualization, Writing - Original Draft, Writing - review & editing. **Sazan Qadir Maulud:** Conceptualization, Data Curation, Visualization, Writing - Original Draft, Writing - review & editing. **Paywast Jamal Jalal**: Writing - Original Draft, Writing - review & editing. **Saman Muhsin Abdulkareem:** Writing - Original Draft, Writing - review & editing. **Jivan Qasim Ahmed**: Writing - Original Draft, Writing - review & editing. **Manish Dhawan:** Writing - Original Draft, Writing - review & editing. **Priyanka:** Writing - Original Draft, Writing - review & editing. **Om Prakash Choudhary:** Supervision, Writing - Original Draft, Writing - review & editing.

## Registration of research studies

1. Name of the registry: Not applicable
2. Unique Identifying number or registration ID: Not applicable
3. Hyperlink to your specific registration (must be publicly accessible and will be checked): Not applicable

## Guarantor

Om Prakash Choudhary, Assistant Professor, Department of Veterinary Anatomy and Histology, College of Veterinary Sciences and Animal Husbandry, Central Agricultural University (I), Selesih, Aizawl-796015, Mizoram, India. Tel: +91–9928099090; Email: dr.om.choudhary@gmail.com.

## Declaration of competing interestCOI

All authors report no conflicts of interest relevant to this article.

## Sources of funding

This study received no specific grant from any funding agency in the public, commercial, or not-for-profit sectors.

## References

[1] Tong J.Y., Wong A., Zhu D., Fastenberg J.H., Tham T. (2020). The prevalence of olfactory and gustatory dysfunction in COVID-19 patients: a systematic review and meta-analysis. Otolaryngol. Head Neck Surg..

[2] Zhou P., Yang X.L., Wang X.G., Hu B., Zhang L., Zhang W., Si H.R., Zhu Y., Li B., Huang C.L., Chen H.D., Chen J., Luo Y., Guo H., Jiang R.D., Liu M.Q., Chen Y., Shen X.R., Wang X., Zheng X.S., Zhao K., Chen Q.J., Deng F., Liu L.L., Yan B., Zhan F.X., Wang Y.Y., Xiao G.F., Shi Z.L. (2020). A pneumonia outbreak associated with a new coronavirus of probable bat origin. Nature.

[3] Mehraeen E., Behnezhad F., Salehi M.A., Noori T., Harandi H., Seyedalinaghi S. (2021). Olfactory and gustatory dysfunctions due to the coronavirus disease (COVID-19): a review of current evidence. Eur. Arch. Oto-Rhino-Laryngol..

[4] Samaranayake L.P., Fakhruddin K.S., Panduwawala C. (2020). Sudden onset, acute loss of taste and smell in coronavirus disease 2019 (COVID-19): a systematic review. Acta Odontol. Scand..

[5] Gui M., Song W., Zhou H., Xu J., Chen S., Xiang Y., Wang X. (2017). Cryo-electron microscopy structures of the SARS-CoV spike glycoprotein reveal a prerequisite conformational state for receptor binding. Cell Res..

[6] Tanasa I.A., Manciuc C., Carauleanu A., Navolan D.B., Bohiltea R.E., Nemescu D. (2020). Anosmia and ageusia associated with coronavirus infection (COVID-19) - what is known?. Exp. Ther. Med..

[7] Dhawan M., Dhama K., Parmar M., Sharma A., Angural S. (2021). Unravelling the potentialities of tocilizumab for the development of a potential immunotherapeutic regimen against COVID-19- A narrative review. J. Appl. Pharmaceut. Sci..

[8] Equils O., Lekaj K., Fattani S., Wu A., Liu G. (2021). Proposed mechanism for anosmia during COVID-19: the role of local zinc distribution. J. Transl. Sci..

[9] Giacomelli A., Pezzati L., Conti F., Bernacchia D., Siano M., Oreni L., Rusconi S., Gervasoni C., Ridolfo A.L., Rizzardini G., Antinori S., Galli M. (2020). Self-reported olfactory and taste disorders in patients with severe acute respiratory coronavirus 2 infection: a cross-sectional study. Clin. Infect. Dis..

[10] Kirchdoerfer R.N., Wang N., Pallesen J., Wrapp D., Turner H.L., Cottrell C.A., Corbett K.S., Graham B.S., Mclellan J.S., Ward A.B. (2018). Stabilized coronavirus spikes are resistant to conformational changes induced by receptor recognition or proteolysis. Sci. Rep..

[11] Hura N., Xie D.X., Choby G.W., Schlosser R.J., Orlov C.P., Seal S.M., Rowan N.R. (2020). Treatment of post-viral olfactory dysfunction: an evidence-based review with recommendations. Int. Forum. Allergy Rhinol..

[12] Rebholz H., Braun R.J., Ladage D., Knoll W., Kleber C., Hassel A.W. (2020). Loss of olfactory function-early indicator for covid-19, other viral infections and neurodegenerative disorders. Front. Neurol..

[13] Speth M.M., Singer-Cornelius T., Oberle M., Gengler I., Brockmeier S.J., Sedaghat A.R. (2020). Olfactory dysfunction and sinonasal symptomatology in COVID-19: prevalence, severity, timing, and associated characteristics. Otolaryngol. Head Neck Surg..

[14] Parma V., Ohla K., Veldhuizen M.G., Niv M.Y., Kelly C.E., Bakke A.J., Cooper K.W., Bouysset C., Pirastu N., Dibattista M., Kaur R., Liuzza M.T., Pepino M.Y., Schöpf V., Pereda-Loth V., Olsson S.B., Gerkin R.C., Rohlfs Domínguez P., Albayay J., Farruggia M.C., Bhutani S., Fjaeldstad A.W., Kumar R., Menini A., Bensafi M., Sandell M., Konstantinidis I., Di Pizio A., Genovese F., Öztürk L., Thomas-Danguin T., Frasnelli J., Boesveldt S., Saatci Ö., Saraiva L.R., Lin C., Golebiowski J., Hwang L.D., Ozdener M.H., Guàrdia M.D., Laudamiel C., Ritchie M., Havlícek J., Pierron D., Roura E., Navarro M., Nolden A.A., Lim J., Whitcroft K.L., Colquitt L.R., Ferdenzi C., Brindha E.V., Altundag A., Macchi A., Nunez-Parra A., Patel Z.M., Fiorucci S., Philpott C.M., Smith B.C., Lundström J.N., Mucignat C., Parker J.K., Van Den Brink M., Schmuker M., Fischmeister F.P.S., Heinbockel T., Shields V.D.C., Faraji F., Santamaría E., Fredborg W.E.A., Morini G., Olofsson J.K., Jalessi M., Karni N., D'errico A., Alizadeh R., Pellegrino R., Meyer P., Huart C., Chen B., Soler G.M., Alwashahi M.K., Welge-Lüssen A., Freiherr J., De Groot J.H.B., Klein H., Okamoto M., Singh P.B., Hsieh J.W., Reed D.R., Hummel T., Munger S.D., Hayes J.E. (2020). More than smell-COVID-19 is associated with severe impairment of smell, taste, and chemesthesis. Chem. Senses.

[15] Al-Zaidi H.M.H., Badr H.M. (2020). Incidence and recovery of smell and taste dysfunction in COVID-19 positive patients. Egypt. J. Otolaryngol..

[16] Hummel T., Landis B.N., Hüttenbrink K.B. (2011). Smell and taste disorders. GMS Curr. Top. Otorhinolaryngol., Head Neck Surg..

[17] Bagheri S.H., Asghari A., Farhadi M., Shamshiri A.R., Kabir A., Kamrava S.K., Jalessi M., Mohebbi A., Alizadeh R., Honarmand A.A., Ghalehbaghi B., Salimi A., Dehghani Firouzabadi F. (2020). Coincidence of COVID-19 epidemic and olfactory dysfunction outbreak in Iran. Med. J. Islam. Repub. Iran.

[18] Printza A., Katotomichelakis M., Valsamidis K., Metallidis S., Panagopoulos P., Panopoulou M., Petrakis V., Constantinidis J. (2021). Smell and taste loss recovery time in COVID-19 patients and disease severity. J. Clin. Med..

[19] Holshue M.L., Debolt C., Lindquist S., Lofy K.H., Wiesman J., Bruce H., Spitters C., Ericson K., Wilkerson S., Tural A., Diaz G., Cohn A., Fox L., Patel A., Gerber S.I., Kim L., Tong S., Lu X., Lindstrom S., Pallansch M.A., Weldon W.C., Biggs H.M., Uyeki T.M., Pillai S.K. (2020). First case of 2019 novel coronavirus in the United States. N. Engl. J. Med..

[20] Gengler I., Wang J.C., Speth M.M., Sedaghat A.R. (2020). Sinonasal pathophysiology of SARS-CoV-2 and COVID-19: a systematic review of the current evidence. Laryngoscope Invest. Otolaryngol.

[21] Maulud S.Q., Majed S.O., Ali A.A., Jalal P.J., Azeez S.H., Mohammad K.A. (2020). Epidemiological approach of SARS-CoV2 in the first month of appearance in the kurdistan region of Iraq. Eur. J. Mol. Clin. Med..

[22] Bratt M., Moen K.G., Nordgård S., Helvik A.S., Skandsen T. (2020). Treatment of posttraumatic olfactory dysfunction with corticosteroids and olfactory training. Acta Otolaryngol..

[23] Hosseini A., Mirmahdi E., Moghaddam M.A. (2020). A new strategy for treatment of Anosmia and Ageusia in COVID-19 patients. Integr. Respir. Med..

[24] Kollndorfer K., Kowalczyk K., Hoche E., Mueller C.A., Pollak M., Trattnig S., Schopf V. (2014). Recovery of olfactory function induces neuroplasticity effects in patients with smell loss. Neural Plast..

[25] Whitman M.C., Greer C.A. (2009). Adult neurogenesis and the olfactory system. Prog. Neurobiol..

[26] Zhao K., Scherer P.W., Hajiloo S.A., Dalton P. (2004). Effect of anatomy on human nasal air flow and odorant transport patterns: implications for olfaction. Chem. Senses.

[27] Van Riel D., Verdijk R., Kuiken T. (2015). The olfactory nerve: a shortcut for influenza and other viral diseases into the central nervous system. J. Pathol..

[28] Attems J., Walker L., Jellinger K.A. (2015). Olfaction and aging: a mini-review. Gerontology.

[29] Glezer I., Malnic B. (2019). Olfactory receptor function. Handb. Clin. Neurol..

[30] Crisafulli U., Xavier A.M., Dos Santos F.B., Cambiaghi T.D., Chang S.Y., Porcionatto M., Castilho B.A., Malnic B., Glezer I. (2018). Topical dexamethasone administration impairs protein synthesis and neuronal regeneration in the olfactory epithelium. Front. Mol. Neurosci..

[31] Han A.Y., Mukdad L., Long J.L., Lopez I.A. (2020). Anosmia in COVID-19: mechanisms and significance. Chem. Senses.

[32] Boesveldt S., Postma E.M., Boak D., Welge-Luessen A., Schöpf V., Mainland J.D., Martens J., Ngai J., Duffy V.B. (2017). Anosmia-A clinical review. Chem. Senses.

[33] Mullol J., Alobid I., Mariño-Sánchez F., Quintó L., De Haro J., Bernal-Sprekelsen M., Valero A., Picado C., Marin C. (2012). Furthering the understanding of olfaction, prevalence of loss of smell and risk factors: a population-based survey (OLFACAT study). BMJ Open.

[34] Jaume F., Quintó L., Alobid I., Mullol J. (2018). Overuse of diagnostic tools and medications in acute rhinosinusitis in Spain: a population-based study (the PROSINUS study). BMJ Open.

[35] Mullol J., Mariño-Sánchez F., Valls M., Alobid I., Marin C. (2020). The sense of smell in chronic rhinosinusitis. J. Allergy Clin. Immunol..

[36] Izquierdo-Dominguez A., Rojas-Lechuga M.J., Mullol J., Alobid I. (2020). Olfactory dysfunction in the COVID-19 outbreak. J Investig. Allergol. Clin. Immunol..

[37] Haehner A., Hummel T., Hummel C., Sommer U., Junghanns S., Reichmann H. (2007). Olfactory loss may be a first sign of idiopathic Parkinson's disease. Mov. Disord..

[38] Ou X., Liu Y., Lei X., Li P., Mi D., Ren L., Guo L., Guo R., Chen T., Hu J., Xiang Z., Mu Z., Chen X., Chen J., Hu K., Jin Q., Wang J., Qian Z. (2020). Characterization of spike glycoprotein of SARS-CoV-2 on virus entry and its immune cross-reactivity with SARS-CoV. Nat. Commun..

[39] Butowt R., Bilinska K. (2020). SARS-CoV-2: olfaction, brain infection, and the urgent need for clinical samples allowing earlier virus detection. ACS Chem. Neurosci..

[40] Hoffmann M., Kleine-Weber H., Schroeder S., Krüger N., Herrler T., Erichsen S., Schiergens T.S., Herrler G., Wu N.H., Nitsche A., Müller M.A., Drosten C., Pöhlmann S. (2020). SARS-CoV-2 cell entry depends on ACE2 and TMPRSS2 and is blocked by a clinically proven protease inhibitor. Cell.

[41] Beltrán-Corbellini Á., Chico-García J.L., Martínez-Poles J., Rodríguez-Jorge F., Natera-Villalba E., Gómez-Corral J., Gómez-López A., Monreal E., Parra-Díaz P., Cortés-Cuevas J.L., Galán J.C., Fragola-Arnau C., Porta-Etessam J., Masjuan J., Alonso-Cánovas A. (2020). Acute-onset smell and taste disorders in the context of COVID-19: a pilot multicentre polymerase chain reaction based case-control study. Eur. J. Neurol..

[42] Brann D.H., Tsukahara T., Weinreb C., Lipovsek M., Van Den Berge K., Gong B., Chance R., Macaulay I.C., Chou H.J., Fletcher R.B., Das D., Street K., De Bezieux H.R., Choi Y.G., Risso D., Dudoit S., Purdom E., Mill J., Hachem R.A., Matsunami H., Logan D.W., Goldstein B.J., Grubb M.S., Ngai J., Datta S.R. (2020). Non-neuronal expression of SARS-CoV-2 entry genes in the olfactory system suggests mechanisms underlying COVID-19-associated anosmia. Sci. Adv..

[43] Moein S.T., Hashemian S.M., Mansourafshar B., Khorram-Tousi A., Tabarsi P., Doty R.L. (2020). Smell dysfunction: a biomarker for COVID-19. Int. Forum. Allergy Rhinol..

[44] Rabaan A.A., Al-Ahmed S.H., Garout M.A., Al-Qaaneh A.M., Sule A.A., Tirupathi R., Mutair A.A., Alhumaid S., Hasan A., Dhawan M., Tiwari R., Sharun K., Mohapatra R.K., Mitra S., Emran T.B., Bilal M., Singh R., Alyami S.A., Moni M.A., Dhama K. (2021 May 7). Diverse immunological factors influencing pathogenesis in patients with COVID-19: a review on viral dissemination, immunotherapeutic options to counter cytokine storm and inflammatory responses. Pathogens.

[45] Vabret N., Britton G.J., Gruber C., Hegde S., Kim J., Kuksin M., Levantovsky R., Malle L., Moreira A., Park M.D., Pia L., Risson E., Saffern M., Salomé B., Esai Selvan M., Spindler M.P., Tan J., Van Der Heide V., Gregory J.K., Alexandropoulos K., Bhardwaj N., Brown B.D., Greenbaum B., Gümüş Z.H., Homann D., Horowitz A., Kamphorst A.O., Curotto De Lafaille M.A., Mehandru S., Merad M., Samstein R.M. (2020). Immunology of COVID-19: current state of the science. Immunity.

[46] Kanjanaumporn J., Aeumjaturapat S., Snidvongs K., Seresirikachorn K., Chusakul S. (2020). Smell and taste dysfunction in patients with SARS-CoV-2 infection: a review of epidemiology, pathogenesis, prognosis, and treatment options. Asian Pac. J. Allergy Immunol..

[47] Dahm T., Rudolph H., Schwerk C., Schroten H., Tenenbaum T. (2016). Neuroinvasion and inflammation in viral central nervous system infections. Mediat. Inflamm..

[48] Mcgavern D.B., Kang S.S. (2011). Illuminating viral infections in the nervous system. Nat. Rev. Immunol..

[49] Chapman C.D., Frey W.H., Craft S., Danielyan L., Hallschmid M., Schiöth H.B., Benedict C. (2013). Intranasal treatment of central nervous system dysfunction in humans. Pharm. Res. (N. Y.).

[50] Li Y.C., Bai W.Z., Hashikawa T. (2020). The neuroinvasive potential of SARS-CoV2 may play a role in the respiratory failure of COVID-19 patients. J. Med. Virol..

[51] Gandhi S., Srivastava A.K., Ray U., Tripathi P.P. (2020). Is the collapse of the respiratory center in the brain responsible for respiratory breakdown in COVID-19 patients?. ACS Chem. Neurosci..

[52] Baig A.M., Khaleeq A., Ali U., Syeda H. (2020). Evidence of the COVID-19 virus targeting the CNS: tissue distribution, host-virus interaction, and proposed neurotropic mechanisms. ACS Chem. Neurosci..

[53] Desforges M., Miletti T.C., Gagnon M., Talbot P.J. (2007). Activation of human monocytes after infection by human coronavirus 229E. Virus Res..

[54] Stegelmeier A.A., Van Vloten J.P., Mould R.C., Klafuric E.M., Minott J.A., Wootton S.K., Bridle B.W., Karimi K. (2019). Myeloid cells during viral infections and inflammation. Viruses.

[55] Russell B., Moss C., Rigg A., Van Hemelrijck M. (2020). COVID-19 and treatment with NSAIDs and corticosteroids: should we be limiting their use in the clinical setting?. E Cancer Med. Sci..

[56] Stenner M., Vent J., Hüttenbrink K.B., Hummel T., Damm M. (2008). Topical therapy in anosmia: relevance of steroid-responsiveness. Laryngoscope.

[57] Lechien J.R., Chiesa-Estomba C.M., De Siati D.R., Horoi M., Le Bon S.D., Rodriguez A., Dequanter D., Blecic S., El Afia F., Distinguin L., Chekkoury-Idrissi Y., Hans S., Delgado I.L., Calvo-Henriquez C., Lavigne P., Falanga C., Barillari M.R., Cammaroto G., Khalife M., Leich P., Souchay C., Rossi C., Journe F., Hsieh J., Edjlali M., Carlier R., Ris L., Lovato A., De Filippis C., Coppee F., Fakhry N., Ayad T., Saussez S. (2020). Olfactory and gustatory dysfunctions as a clinical presentation of mild-to-moderate forms of the coronavirus disease (COVID-19): a multicenter European study. Eur. Arch. Oto-Rhino-Laryngol..

[58] Guan W.J., Ni Z.Y., Hu Y., Liang W.H., Ou C.Q., He J.X., Liu L., Shan H., Lei C.L., Hui D.S.C., Du B., Li L.J., Zeng G., Yuen K.Y., Chen R.C., Tang C.L., Wang T., Chen P.Y., Xiang J., Li S.Y., Wang J.L., Liang Z.J., Peng Y.X., Wei L., Liu Y., Hu Y.H., Peng P., Wang J.M., Liu J.Y., Chen Z., Li G., Zheng Z.J., Qiu S.Q., Luo J., Ye C.J., Zhu S.Y., Zhong N.S. (2020). Clinical characteristics of coronavirus disease 2019 in China. N. Engl. J. Med..

[59] Ceccarelli M., Berretta M., Venanzi Rullo E., Nunnari G., Cacopardo B. (2020). Differences and similarities between Severe Acute Respiratory Syndrome (SARS)-CoronaVirus (CoV) and SARS-CoV-2. Would a rose by another name smell as sweet?. Eur. Rev. Med. Pharmacol. Sci..

[60] Hummel T., Stupka G., Haehner A., Poletti S.C. (2018). Olfactory training changes electrophysiological responses at the level of the olfactory epithelium. Rhinology.

[61] Andreini C., Banci L., Bertini I., Rosato A. (2006). Counting the zinc-proteins encoded in the human genome. J. Proteome Res..

[62] Te Velthuis A.J., Van Den Worm S.H., Sims A.C., Baric R.S., Snijder E.J., Van Hemert M.J. (2010). Zn (2+) inhibits coronavirus and arterivirus RNA polymerase activity in vitro and zinc ionophores block the replication of these viruses in cell culture. PLoS Pathog..

